# Prevalence of central nervous system-active polypharmacy in a cohort of older adults in Argentina

**DOI:** 10.1192/bjo.2024.798

**Published:** 2024-10-29

**Authors:** Augusto Ferraris, Federico Angriman, Tomas Barrera, Paula Penizzotto, Sol Faerman, Washington Rivadeneira, Alan Chiessa, Gaspar Mura, Javier Alberto Pollán, Alejandro G. Szmulewicz

**Affiliations:** Department of Epidemiology, School of Public Health, University of Washington, Seattle, USA; and Laboratory of Applied Statistics in Health Sciences, School of Medicine, University of Buenos Aires, Buenos Aires, Argentina; Department of Critical Care Medicine, Sunnybrook Health Sciences Centre, Toronto, Canada; and Interdepartmental Division of Critical Care Medicine, University of Toronto, Toronto, Canada; Laboratory of Applied Statistics in Health Sciences, School of Medicine, University of Buenos Aires, Buenos Aires, Argentina; and Department of Internal Medicine, Hospital Italiano de Buenos Aires, Buenos Aires, Argentina; Department of Internal Medicine, Hospital Italiano de Buenos Aires, Buenos Aires, Argentina; Epidemiology Department, Harvard TH Chan School of Public Health, Boston, USA

**Keywords:** Epidemiology, central nervous system, polypharmacy, geriatrics, mental health

## Abstract

**Background:**

Central nervous system (CNS)-active polypharmacy is frequent and potentially harmful in older patients. Data on its burden outside the USA and European countries remain limited.

**Aims:**

To estimate the period prevalence of and factors associated with out-of-hospital CNS-active polypharmacy in older adults.

**Method:**

We used data from a cohort of out-patients aged ≥60 years affiliated to the Hospital Italiano de Buenos Aires’ health maintenance organisation on 1 January 2021. A CNS-active polypharmacy event was defined as the concurrent exposure to ≥3 CNS-active medications (i.e. antidepressants, anti-epileptics, antipsychotics, benzodiazepines, Z-drugs and opioids) through filled out-of-hospital prescriptions. We calculated the period prevalence of CNS-active polypharmacy for 2021. We identified factors associated with CNS-active polypharmacy using a multivariable logistic regression model to estimate odds ratios and 95% confidence intervals (CI).

**Results:**

We included 63 857 patients. Pre-existing mental health diagnoses included anxiety (21%), depressive (14%) and sleep (11%) disorders. CNS-active polypharmacy occurred in 4535 patients, for a period prevalence of 7.1% (95% CI: 6.9–7.3%). The combination of an antidepressant, an antipsychotic and a benzodiazepine accounted for 21% of the CNS-active polypharmacy events. Frontotemporal dementia (odds ratio: 14.67; 95% CI: 4.47–48.20), schizophrenia (odds ratio: 7.93; 95% CI: 4.64–13.56), bipolar disorder (odds ratio: 7.20; 95% CI: 5.45–9.50) and depressive disorder (odds ratio: 3.50; 95% CI: 3.26–3.75) were associated with CNS-active polypharmacy.

**Conclusions:**

One in 14 adults aged 60 years and older presented out-of-hospital CNS-active polypharmacy. Future studies should evaluate measures to reduce CNS-active medication use in this population.

Potentially inappropriate drug prescribing and polypharmacy in older patients can lead to drug–drug interactions and increased risk of drug-related adverse events.^1^ The use of multiple, concurrent medications that act in the central nervous system (CNS) can have larger impacts on older patients because of physiologic changes related to age (e.g. enhanced CNS penetration, decreased metabolisation).^2^ For instance, prescribers should avoid the use of concurrent CNS-active medications in older patients because of their association with a higher risk of falls and accelerated cognitive decline.^3,4^ Further, observational studies have reported that patients with CNS-active polypharmacy use present with a higher risk of cardiovascular events, unintentional overdoses, admissions to hospital and all-cause mortality compared with those without.^4–8^ While the underpinnings of the associations described for CNS-active polypharmacy and these adverse events remain to be completely elucidated, current evidence suggests that such medications are detrimental in older adults and their use should be minimised whenever possible.

## Current evidence

Despite formal recommendations against the use of CNS-active polypharmacy in older individuals (e.g. Beers criteria and a black box warning for concurrent use of opioids with benzodiazepines, Z-drugs and other CNS depressants),^9,10^ recent evidence suggests that CNS-active polypharmacy remains frequent.^11–13^ Notably, available data on the burden of CNS-active polypharmacy are limited to populations from the USA and Europe.^11–13^ Medication use may diverge in Latin American countries such as Argentina, but studies measuring CNS-active polypharmacy are lacking. The healthcare system of Argentina differs from those of the USA and Europe, as it is characterised by the intersection of three sectors: (a) the state-funded public healthcare sector, which provides coverage mainly to uninsured individuals (40–45% of the population); (b) the social system healthcare sector, which provides labour union-run insurance services to formal workers (50–55%); and (c) the private sector, which includes both for-profit and not-for-profit organisations providing care to their clients (5–8%).^14^ Generally, patients in the private healthcare sector have a higher income, while fees for medication purchases are also higher than in the public and social sectors.^14^ Hence, patterns of CNS-active medication use in this setting may differ compared with the reports of previous studies.^11–13^ Understanding the burden of CNS-polypharmacy and its drivers in diverse populations thus remains crucial to identify effective strategies to help providers avoid CNS-polypharmacy altogether and deploy safe deprescribing interventions.

## Objectives

We conducted a retrospective cohort study to estimate the prevalence of out-of-hospital CNS-active polypharmacy among individuals aged 60 years or older in a health maintenance organisation in Argentina. Our objectives were to (a) describe CNS-active polypharmacy period prevalence during 2021 and (b) investigate sociodemographic and clinical factors associated with CNS-active polypharmacy.

## Method

### Data sources

The present cohort study was conducted using out-of-hospital, individual-patient-level data from the Hospital Italiano de Buenos Aires’ health maintenance organisation collected between 1 January 2017 and 31 December 2021. The hospital is one of the largest university teaching hospitals in Argentina and Latin America and it is accredited by the Joint Commission International.^15^ The institution manages its own private, not-for-profit, healthcare network, which provides care to over 170 000 affiliates in Argentina, with an extensive system of pharmacies, out-patient services and low- and high-complexity facilities (e.g. two university teaching hospitals, numerous medical offices).^16^ The Hospital Italiano de Buenos Aires’ health maintenance organisation uses integrated electronic health records that gather information on out-of-hospital and in-hospital diagnosis and procedures using SNOMED CT, Spanish 2020 edition for Windows (SNOMED international, London, UK; see https://www.snomed.org).^17^ Healthcare professionals input clinical terminologies into electronic health records, and SNOMED CT encodes these terminologies using standardised terms that are shared across medical specialties and healthcare systems.^17^ Finally, the health maintenance organisation has a comprehensive registry of medication purchases of patients. Filled prescriptions are encoded using the Anatomical Therapeutic Chemical (ATC) index codes and captures patients’ identifier, milligrams per pill, number of pills and number of boxes purchased.^18^ However, the database does not capture the days of supply or the clinical indication for the filled prescription.

### Study population and study period

Eligible individuals were aged 60 years or older on 1 January 2021 (index date) and affiliated to the Hospital Italiano de Buenos Aires’ health maintenance organisation. Patients were followed from the index date until disaffiliation from the health maintenance organisation, death or end of the study period (31 December 2021).

### CNS-active polypharmacy definition

CNS-active medications were defined as any out-of-hospital filled prescription for an agent from the following medication classes: anti-epileptics, benzodiazepines, Z-drugs, antipsychotics, opioids and antidepressants (i.e. selective serotonin receptor inhibitors, serotonin–noradrenaline reuptake inhibitors and tricyclic antidepressants)^19^ (Supplementary Table 1 available at https://doi.org/10.1192/bjo.2024.798). A CNS-active polypharmacy event was defined as the concurrent exposure to three or more CNS-active medications. We considered patients exposed to a CNS-active medication through the month of prescription fill and an additional period of 1 month after the prescription was not refilled to account for real-world practices in medication use (i.e. gaps in medication refilling such as stockpiling and variations in medication purchase practices).^20,21^

There were no restrictions to the number of medications classes or combinations of medication classes involved in CNS-active polypharmacy. For example, one antipsychotic, one Z-drug and one benzodiazepine; two distinct antidepressants and one antipsychotic; or three distinct benzodiazepines could all be counted as a CNS-active polypharmacy event.^11,19^

### Additional covariates

We captured baseline information (i.e. at index date) on age, gender, comorbidities (i.e. hypertension, cardiac failure, coronary heart disease, peripheral vascular disease, diabetes mellitus, chronic kidney disease, cirrhosis, cancer, chronic obstructive pulmonary disease, asthma, tobacco use, alcohol misuse, cognitive complaint, dementia and its subtypes, Parkinson's disease, anxiety and depressive disorders, bipolar disease, schizophrenia, sleep disorders and epilepsy) and the number of admissions to hospital in the preceding year at the individual patient level.

To estimate the positive predictive value of the clinical diagnoses, we reviewed the electronic medical charts of a probabilistic sample of each diagnosis (Supplementary Table 2). Five investigators (A.C., W.R., S.F., P.P., G.M.) reviewed the charts and recorded whether the diagnosis captured by the SNOMED CT term matched clinical criteria for the disease. Thus, we estimated the positive predictive value of each condition by calculating the number of patients classified by the medical doctors as true cases over the total number of patients classified by SNOMED CT terms. Any discrepancies were resolved by the lead author (A.F.).

### Statistical analysis

To describe the CNS-active polypharmacy period prevalence during 2021, we calculated the sum of individuals with CNS-active polypharmacy: (a) on 1 January 2021 (i.e. prevalent users of CNS-active polypharmacy) and (b) during the study period (from 1 January 2021 to 31 December 2021) (i.e. incident users of CNS-active polypharmacy), divided by the total number of individuals in the cohort on 1 January 2021.^20^ We constructed 95% confidence intervals using the methods described by Clopper and Pearson (exact confidence intervals).^22^

To identify potential factors associated with CNS-active polypharmacy at the individual patient level, we used a multivariable logistic regression model to estimate odds ratios and 95% confidence intervals. Variables included in the exploratory model were demographics (i.e. age, gender), mental health diagnoses (i.e. anxiety and depressive disorders, sleep disorders, schizophrenia, bipolar disorder), neurodegenerative disorders (i.e. cognitive complaint, Parkinson's disease, Alzheimer's disease, vascular, frontotemporal, Lewy body or any type of dementia), other comorbidities (i.e. number of prior admissions to hospital in the preceding year as a continuous variable, epilepsy, hypertension, cardiac failure, coronary disease, peripheral vascular disease, diabetes mellitus, chronic kidney disease, cirrhosis, cancer, chronic obstructive pulmonary disease, asthma) and substance use (i.e. past or current alcohol misuse and tobacco use). Thus, for example, the estimated odds ratio of the exploratory multivariable model compared the odds of CNS-active polypharmacy for individuals with depressive disorders at baseline with the odds for those without, while keeping constant the remaining covariables included in the model. Furthermore, we conducted bivariate analyses for the individual association of variables included in the exploratory model and CNS-active polypharmacy.

To identify groups and combinations of drugs driving CNS-active polypharmacy, we described the individual medications, medication classes and combinations of medication classes more frequently involved in CNS-active polypharmacy. We allowed for multiple events of polypharmacy per patient, such that we recorded the individual medications and medication classes involved in each CNS-active polypharmacy event (Supplementary Table 1).

### Sensitivity and subgroup analyses

To test the robustness of our period-prevalence analysis, we modified our outcome definition of CNS-active polypharmacy as having filled three or more concurrent CNS-active medications: (a) on the same calendar week (without using an additional gap period of 1 month after a prescription was not refilled) and (b) extending the gap period to 2 months after a prescription was not refilled.^21^ Further, since CNS-active medications may be especially deleterious in patients with increasing age and in those with dementia, we conducted subgroup analyses to estimate the period prevalence of CNS-active polypharmacy by diagnosis (patients with and without dementia) and age group (older than 80 years; 70–79; 60–69).^1,2^

The authors assert that all procedures contributing to this work comply with the ethical standards of the relevant national and institutional committees on human experimentation and with the Helsinki Declaration of 1975, as revised in 2008. All procedures involving human participants/patients were approved by institutional review board (Comité de Ética para Protocolos de Investigación del Hospital Italiano de Buenos Aires, protocol number: 7235), and patient consent was waived. The present report followed the REporting of studies Conducted using Observational Routinely-collected Data for PharmacoEpidemiology (RECORD-PE) statements for pharmacoepidemiologic studies.^23^ All analyses were performed using R statistical software for Windows, version 4.3.1 (R Foundation for Statistical Computing, Vienna, Austria; see https://www.r-project.org).

## Results

**Figure 1:**
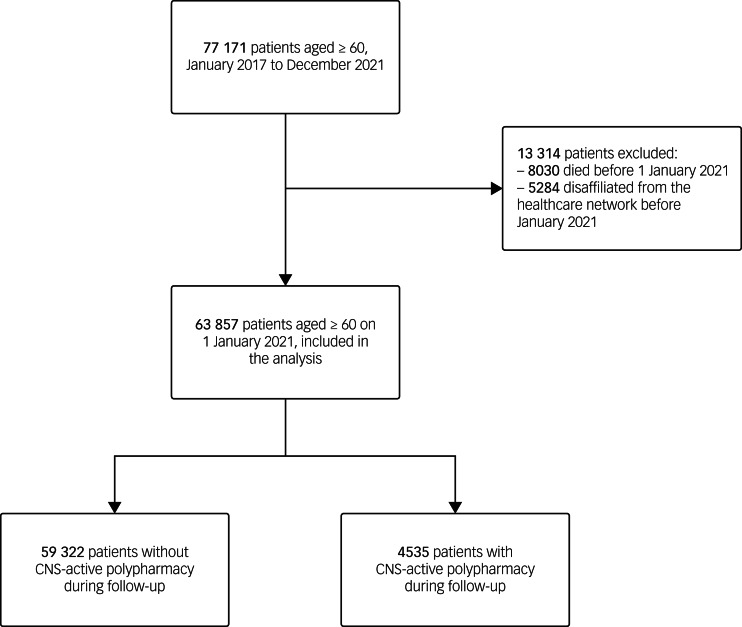
Flowchart of study patients. CNS, central nervous system.

Of the initial 77 171 patients available in the cohort, 63 857 were included in the present study (Fig. 1). Overall, 443 (0.7%) patients disaffiliated from the health maintenance organisation and 2332 (3.7%) patients died during follow-up. Baseline characteristics of patients included are summarised in Table 1. The cohort mainly comprised individuals aged 70 years or older and most patients were female. Anxiety (21%), depressive (14%) and sleep (11%) disorders were the most frequent mental health diagnoses in the entire cohort at baseline. Hypertension was the most frequent comorbid condition and roughly a fifth of the patients presented either current or previous tobacco use (Table 1).

**Table 1:** Characteristics of adults aged 60 years and older on 1 January 2021 (Hospital Italiano de Buenos Aires, 2021)

Characteristics	All participants (*N* = 63 857)	
	*n*	%
Demographics		
Age group, in years		
60–69	18 283	28
70–79	22 767	36
80 or more	22 807	36
Female	42 110	66
Mental health diagnoses		
Anxiety disorder	13 575	21
Depressive disorder	8914	14
Sleep disorder	7189	11
Epilepsy	829	1
Bipolar disease	275	<1
Schizophrenia	74	<1
Neurodegenerative diseases		
Cognitive complaint	4316	7
Dementia, any type	1700	3
Alzheimer's disease	351	1
Vascular dementia	109	<1
Frontotemporal dementia	<20	<1
Lewy body dementia	127	<1
Parkinson's disease	492	1
Other comorbidities		
Hypertension	41 145	64
Cardiac failure	2810	4
Coronary disease	5146	8
Peripheral vascular disease	1427	2
Diabetes mellitus	7685	12
Chronic kidney disease	3075	5
Cirrhosis	712	1
Cancer	7802	12
COPD	3030	5
Asthma	3660	6
Alcohol misuse (past or current	819	1
Tobacco use (past or current	13 508	21
Hospital admissions in the previous year		
One admission	4783	8
Two admissions or more	1219	2

COPD, chronic obstructive pulmonary disease.

Frequencies lower than 20 are shown as inequalities.

A total of 4535 individuals had CNS-active polypharmacy during 2021, for a period prevalence of 7.1% (95% CI: 6.9–7.3%). Of these, 1211 were prevalent users of CNS-active polypharmacy (Supplementary Figure 1). Prevalent users of CNS-active polypharmacy at baseline were on average older, more likely to be female and presented a higher prevalence of mental health, neurodegenerative and cardiovascular and respiratory diseases than those without use of CNS-active polypharmacy (Supplementary Table 3).

### Sensitivity and subgroup analyses

The period prevalence of CNS-active polypharmacy was consistent when we changed our outcome definition. The period prevalence of CNS-active polypharmacy was 5.0% (95% CI: 4.9–5.2%) when we defined polypharmacy as filling prescriptions for three or more CNS-active medications on the same week without a gap period (Table 2). Conversely, when we extended the gap period to 2 months of exposure after a prescription was not refilled, the period prevalence of CNS-active polypharmacy was 7.6% (95% CI: 7.4–7.8%) (Table 2). Finally, among the pre-specified groups of interest, the period prevalence of CNS-active polypharmacy was more than four times higher in individuals with dementia compared with those without. The period prevalence of CNS-active polypharmacy was more than two times higher in individuals aged 80 years or older compared with the prevalence observed in patients aged 60–69 years (Table 2). Conversely, the prevalence of CNS-active polypharmacy in individuals aged 70–79 years was similar to that observed in patients aged 60–69 years (Table 2).

**Table 2:** Prevalence of central nervous system (CNS)-active medications according to different measurement strategies and subgroups of patients

Strategy	Prevalence of CNS-active polypharmacy			
	*N* of events	*N*	%	95% CI^a^
Sensitivity analyses				
Main analysis	4535	63 857	7.1	(6.9–7.3)
Gap period of 2 months^b^	4831	63 857	7.6	(7.4–7.8)
No gap period, same week^c^	3212	63 857	5.0	(4.9–5.2)
Subgroup analyses				
Dementia	520	1700	30.6	(28.4–32.8)
No dementia	4015	62 157	6.5	(6.3–6.7)
Age 60–69	808	18 283	4.4	(4.1–4.7)
Age 70–79	1413	22 767	6.2	(5.9–6.5)
Age ≥80	2314	22 807	10.1	(9.8–10.5)

a. We estimated 95% confidence intervals using Clopper–Pearson (exact) approach for a binomial.

b. Using a gap period of 1 month after a prescription was filled (main analysis) to estimate CNS-active polypharmacy.

c. Counting only prescriptions occurring within the same week, without a gap period to estimate CNS-active polypharmacy.

### Factors associated with CNS-active polypharmacy

Several baseline characteristics were associated with CNS-active polypharmacy (Table 3). We found the largest association between CNS-active polypharmacy with frontotemporal dementia, but our estimates were imprecise. Similarly, severe mental illnesses (i.e. bipolar disorder, major depression and schizophrenia) and a diagnosis of cognitive complaint were positively associated, while cirrhosis was inversely associated with the prevalence of CNS-active polypharmacy (Table 3).

**Table 3:** Baseline factors associated with central nervous system (CNS)-active medication polypharmacy

Baseline variable	CNS-active polypharmacy prevalence (%)	Bivariate model^a^		Multivariable model^b^	
		Odds ratio	95% CI	Odds ratio	95% CI
Age category^c^					
60–69 years	4.4	Ref.	Ref.	Ref.	Ref.
70–79 years	6.2	1.43	(1.31–1.56)	1.17	(1.07–1.29)
80 years and older	10.1	2.44	(2.25–2.65)	1.54	(1.40–1.70)
Gender					
Male	4.5	Ref.	Ref.	Ref.	Ref.
Female	8.4	1.94	(1.81–2.09)	1.52	(1.40–1.65)
Anxiety disorders	13.8	2.86	(2.68–3.04)	1.98	(1.85–2.12)
Depressive disorder	21.9	5.66	(5.31–6.03)	3.50	(3.26–3.75)
Sleep disorder	15.9	2.98	(2.77–3.20)	1.98	(1.83–2.14)
Epilepsy	18.2	2.98	(2.49–3.56)	2.21	(1.72–2.82)
Bipolar disorder	52.4	14.82	(11.67–18.81)	7.20	(5.45–9.50)
Schizophrenia	40.5	8.97	(5.64–14.28)	7.93	(4.64–13.56)
Cognitive complaint	23.7	4.94	(4.57–5.34)	2.66	(2.42–2.92)
Alzheimer's disease	27.6	5.08	(4.02–6.44)	1.45	(1.10–1.93)
Vascular dementia	31.2	5.97	(3.98–8.96)	**1.16**	**(0.72–1.87)**
Frontotemporal dementia	75.0	39.34	(12.68–122.00)	14.67	(4.47–48.20)
Lewy body dementia	40.9	9.16	(6.43–13.07)	2.52	(1.69–3.76)
Parkinson's disease	20.7	3.48	(2.79–4.33)	2.53	(1.98–3.24)
Cancer	9.5	1.46	(1.34–1.58)	1.28	(1.17–1.40)
Cardiac failure	14.1	2.25	(2.02–2.51)	1.21	(1.06–1.38)
Hypertension	7.9	1.41	(1.32–1.50)	**1.02**	**(0.95–1.10)**
Coronary disease	9.1	1.34	(1.21–1.48)	**1.03**	**(0.92–1.17)**
COPD	13.4	2.13	(1.91–2.37)	1.50	(1.31–1.71)
Asthma	9.9	1.48	(1.33–1.66)	**1.09**	**(0.95–1.25)**
Diabetes mellitus	8.4	1.24	(1.14–1.35)	**1.08**	**(0.98–1.19)**
Chronic kidney disease	10.2	1.52	(1.35–1.72)	**1.04**	**(0.90–1.20)**
Cirrhosis	10.7	1.57	(1.24–2.00)	0.38	(0.27–0.55)
Alcohol misuse (past or current)	13.4	2.06	(1.68–2.52)	1.48	(1.18–1.86)
Tobacco use (past or current)	9.0	1.40	(1.31–1.50)	1.13	(1.04–1.22)
Hospital admissions in the prior year^d^	–	1.64	(1.57–1.72)	1.40	(1.33–1.47)

COPD, chronic obstructive pulmonary disease.

a. Bivariate analyses for the association of the listed baseline variables (individually) and central nervous system-active polypharmacy.

b. Multivariable logistic regression model with 95% confidence intervals estimated using Wald's formula.

c. The reported odds ratio used indicator variable coding to compare age categories 70–79 and 80 years and older with the reference group of 60–69 years. Odds ratios were estimated including all age categories using indicator variable coding.

d. Hospital admission was included in the model as a discrete variable, and the reported odds ratio compared the odds of central nervous system-active polypharmacy of two groups of individuals that differ by one hospital admission in the preceding year, with individuals with a higher number of admissions to hospital presenting a higher odds of central nervous system-active polypharmacy.

Statistically non-significant associations at the 0.05 level for the multivariable analysis are showed in bold format.

### Prevalence of CNS-active medication, medication classes and individual medications

A total of 24 596 CNS-active polypharmacy events that involved 80 775 CNS-active medications were recorded among 4535 patients with CNS-active polypharmacy during follow-up. The median time from index date until the first CNS-active polypharmacy event was 3 (interquartile range, 0–7) months (Supplementary Figure 1). Antidepressant, benzodiazepine and antipsychotic classes accounted for 72% of the total prescriptions involved in CNS-active polypharmacy events. Overall, antidepressants were present in 9 of the 10 most frequent medication classes combinations involved in CNS-active polypharmacy events (Table 4). The most frequent combinations of medication classes were as follows: an antidepressant, an antipsychotic and a benzodiazepine in 21% of CNS-active polypharmacy events; an antidepressant, an anti-epileptic and a benzodiazepine in 9% of CNS-active polypharmacy events; and an antidepressant, a benzodiazepine and a Z-drug in 8% of CNS-active polypharmacy events (Table 4). Finally, medications most frequently involved in CNS-active polypharmacy events were clonazepam (12.9% of prescriptions), quetiapine (11.1% of prescriptions), alprazolam (6.5% of prescriptions), pregabalin (6.3% of prescriptions) and escitalopram (6.2% of prescriptions) (Supplementary Table 4).

**Table 4:** Frequency of medication classes and combinations of medication classes involved in 24 596 central nervous system-active polypharmacy events

Combination of medication classes^a^	Frequency	%
1. Antidepressant, antipsychotic and benzodiazepine	5072	20.6
2. Antidepressant, anti-epileptic and benzodiazepine	2274	9.2
3. Antidepressant, benzodiazepine and Z-drug	1854	7.5
4. Antidepressants and benzodiazepines	1433	5.8
5. Antidepressant, antipsychotic, benzodiazepine and anti-epileptic	1147	4.7
6. Antidepressant, benzodiazepine and opioid	1137	4.6
7. Antidepressant, antipsychotic and anti-epileptic	947	3.9
8. Antidepressants and antipsychotics	845	3.4
9. Antipsychotic, anti-epileptic and benzodiazepine	824	3.4
10. Antidepressant, antipsychotic, benzodiazepine and Z-drug	749	3.0
11. Anti-epileptic, benzodiazepine and opioid	673	2.7
12. Antipsychotics and benzodiazepines	640	2.6
13. Antidepressant, antipsychotic and Z-drug	559	2.3
14. Antidepressant, anti-epileptic, benzodiazepine and opioid	507	2.1
15. Antipsychotic, benzodiazepine and Z-drug	462	1.9
16. Antipsychotic, anti-epileptic and opioid	410	1.7
17. Antidepressant, anti-epileptic and Z-drug	403	1.6
18. Antidepressants and anti-epileptics	331	1.3
19. Antidepressant, anti-epileptic, benzodiazepine and Z-drug	311	1.3
20. Anti-epileptic, benzodiazepine and Z-drug	239	1.0
21. Benzodiazepine, Z-drug and opioids	238	0.9
22. Anti-epileptics and benzodiazepines	233	0.9
23. Benzodiazepines and opioids	202	0.8
24. Antidepressants and Z-drugs	196	0.9
25. Antipsychotic, benzodiazepine and opioid	194	0.8
26. Antidepressant, anti-epileptic, antipsychotics and Z-drug	193	0.8
27. Antidepressant, antipsychotic, benzodiazepine and opioid	179	0.7
28. Anti-epileptics and antipsychotics	172	0.7
29. Antidepressant, antipsychotic and opioid	168	0.7
30. Antidepressant, anti-epileptic, antipsychotic, benzodiazepine and Z-drug	166	0.7
Other combinations (34)	1838	7.4

a. Frequencies and percentage estimates are based on a total of 80 775 individual medications used in 24 596 central nervous system-active polypharmacy events among 4535 patients with central nervous system-active polypharmacy. Percentages may not add age exactly 100% owing to rounding.

## Discussion

The present study evaluated the prevalence and factors associated with CNS-active polypharmacy in a cohort of adults aged 60 years or older in a health maintenance organisation in Argentina. We found that one in 14 adults aged 60 years or older had CNS-active polypharmacy during 2021. The year 2021 avoided the secular influence of the COVID-19 pandemic onset in our estimations, which presented specific challenges to accessing healthcare among older adults in Argentina.^25^ In addition, we provided the most recent estimate of CNS-active polypharmacy available in the cohort. Our estimation of CNS-active polypharmacy burden remained consistent in multiple sensitivity analyses. We also found a higher prevalence of CNS-active polypharmacy in groups of interest, such as patients aged 80 or older and patients with a diagnosis of dementia. This study provides insight into the concurrent use of CNS-active medications in real-world settings and represents a first step towards understanding the drivers of CNS-active polypharmacy in Latin American populations. Further evidence is required to identify groups of patients that may benefit from the implementation of potential deprescribing interventions adapted to the particular characteristics of the region.^1,26^

Previous studies evaluated CNS-active medication use across several populations. In the USA, a 2017 study found a prevalence of CNS-active polypharmacy (defined as the use of three or more CNS-active medications simultaneously) of 1.4% among out-patients aged 65 or older.^12^ The study by Maust et al^12^ reported a noticeably lower prevalence of polypharmacy as compared with our study. Reasons for this discrepancy include differences in (a) analytical approach (i.e. week versus annual period prevalence), (b) medication use ascertainment (i.e. physician's reports versus pharmacy claims), (c) comorbidity burden (i.e. a higher prevalence of anxiety, sleep and depressive disorders in this study population) and (d) healthcare system characteristics (i.e. possibly better access to medications in our study population). In addition, a 2021 study found a prevalence of CNS-active polypharmacy (defined as three or more CNS-active medications for 30 consecutive days or longer) of nearly 14% among patients with a diagnosis of dementia.^11^ In Denmark, a 2016 study reported a prevalence (defined as the concurrent use of two or more CNS-active medication classes) of roughly 25% among patients with dementia.^27^ These findings underscore how, despite variations in the definition of polypharmacy,^28^ CNS-active medication use remained consistently high across different settings. Our study expands the literature by providing estimates from a health maintenance organisation in Argentina.

We also explored clinical factors related to CNS-active polypharmacy. The largest associations were found for mental health disorders that frequently present with psychotic features, that is, frontotemporal dementia, bipolar disorder and schizophrenia. However, our estimates were imprecise because of the low number of cases observed and should be interpreted with caution. More prevalent conditions, such as anxiety and depressive disorders, were also associated with CNS-active polypharmacy. Our findings suggest that older adults with mental health conditions may represent an attractive target to direct efforts to reduce the prescribing of multiple concomitant CNS-active agents. These patients are also at a heightened risk of poor clinical outcomes, including opioid-related overdoses,^6,7^ accelerated cognitive decline, higher risk of falls and higher risk of all-cause mortality.^3,5,29,30^ Previous interventions within this same health maintenance organisation have used alarms set in the electronic health records to identify patients at high risk of poor clinical outcomes to improve their clinical trajectories within the health system network.^31^ A similar approach using risk factors of CNS-active polypharmacy could be implemented, for example, by creating a warning when initiation of treatments would lead to CNS-active polypharmacy in older adults with dementia. Thus, tailored deprescribing interventions^26^ for these patients and their caregivers^32^ could potentially have a large clinical impact. In addition, minimising the duration of CNS-active polypharmacy when its use is required should also be considered. Monitoring the appropriate duration of CNS-active treatments could be an initial measure to reduce CNS-active medication burden, especially when treatments are intended for short-term use (e.g. benzodiazepines).^33^ Until the results of ongoing high-quality randomised clinical trials evaluating deprescribing interventions for CNS-active medications on broader populations are published,^34^ resource allocation to patients at the highest risk of adverse clinical outcomes (e.g. patients in nursing home residencies) could also be considered.^24,35^

This study presents several limitations. First, we used medication purchases documented in out-of-hospital settings to measure the occurrence of CNS-active polypharmacy. While misclassification of CNS-active medication use may still be possible, we expected to capture all out-of-hospital medication purchase events, since the medications included in the present study are unavailable over the counter, require reports to the national regulatory authorities upon purchase and clients have re-imbursement benefits when using the hospital's pharmacy network.^16^ Similarly, we could not identify musculoskeletal relaxant use, a medication class recently added to the CNS-active polypharmacy definition.^9^ For both reasons, our calculations may be a conservative estimate of the true CNS-active polypharmacy prevalence under the new definitions in this setting. Second, we calculated the period prevalence, a measure of disease occurrence that includes both prevalent and incident CNS-active medication users. The inclusion of prevalent CNS-active polypharmacy users casts doubt on causality claims with respect to the association between predictors and polypharmacy.^20^ However, our analyses were exploratory and focused on identifying groups of individuals with high prevalence of CNS-active medication use that may benefit from deprescribing interventions. Furthermore, these groups of patients can be subject of future research to evaluate the impact of such extensive CNS-active polypharmacy exposure on health outcomes. Third, we did not have information on days of supply and we could not evaluate the duration of CNS-active polypharmacy, which may be related to the occurrence of adverse events. Finally, our study was conducted in a health maintenance organisation, and our findings may not be representative of the national or regional trends in CNS-active polypharmacy occurrence. Future studies reproducing our methods in populations with dissimilar health-seeking behaviours, burden of comorbidities and distribution of social determinants of health remain warranted.

In conclusion, one in 14 individuals aged 60 or older presented CNS-active polypharmacy during follow-up in a large health maintenance organisation in Argentina in 2021. CNS-active polypharmacy use was found to be higher in patients aged 80 years or older and in patients with a diagnosis of dementia. Our findings underscore the need for reducing concurrent CNS-active medication use in older patients whenever feasible.

## Supporting information

Ferraris et al. supplementary material Ferraris et al. supplementary material

## Data Availability

The data-set from this study is held securely in coded form. The data are not publicly available because of the presence of sensitive information that could compromise the privacy of research participants. Data sharing agreements from the Hospital Italiano de Buenos Aires prohibit making the data-set publicly available. Furthermore, the creation of this data-set involved the use of several data sources from administrative records that were linked at the individual patient level. While the final data-set cannot be shared, the full data-set creation plan and underlying analytic code are available from the main author (A.F.) upon request.
