# Supplementary material for: Prevalence of central nervous system-active polypharmacy in a cohort of older adults in Argentina
Source: BJPsych Open. 2024 Oct 29;10(6):e190. doi: 10.1192/bjo.2024.798 (PMC11698161; doi:10.1192/bjo.2024.798)
Supplement: Ferraris et al. supplementary material [file S2056472424007981sup001.docx]

**Prevalence of central nervous system-active polypharmacy in a cohort of older adults in Argentina.**

**Supplementary appendix**

**Figure S1.** Month of the first Central Nervous System-active polypharmacy recorded among 4,535 exposed individuals in a health maintenance organization in Argentina, 2021

**Table S1.** Anatomic Therapeutic Chemical codes for Central nervous System-active medications.

**Table S2.** Positive predictive value of SNOMED CT terms based on auditing of random samples of 100 patients for each diagnosis.

**Table S3.** Characteristics of adults aged 60 and older, according to their Central Nervous System-active medication exposure on January 1^st^, 2021 (Hospital Italiano de Buenos Aires).

**Table S4.** Individual drugs involved in 24,596 Central Nervous System-active polypharmacy events.

**Figure S1.** Month of the first Central Nervous System-active polypharmacy recorded among 4,535 exposed individuals in a health maintenance organization in Argentina, 2021.


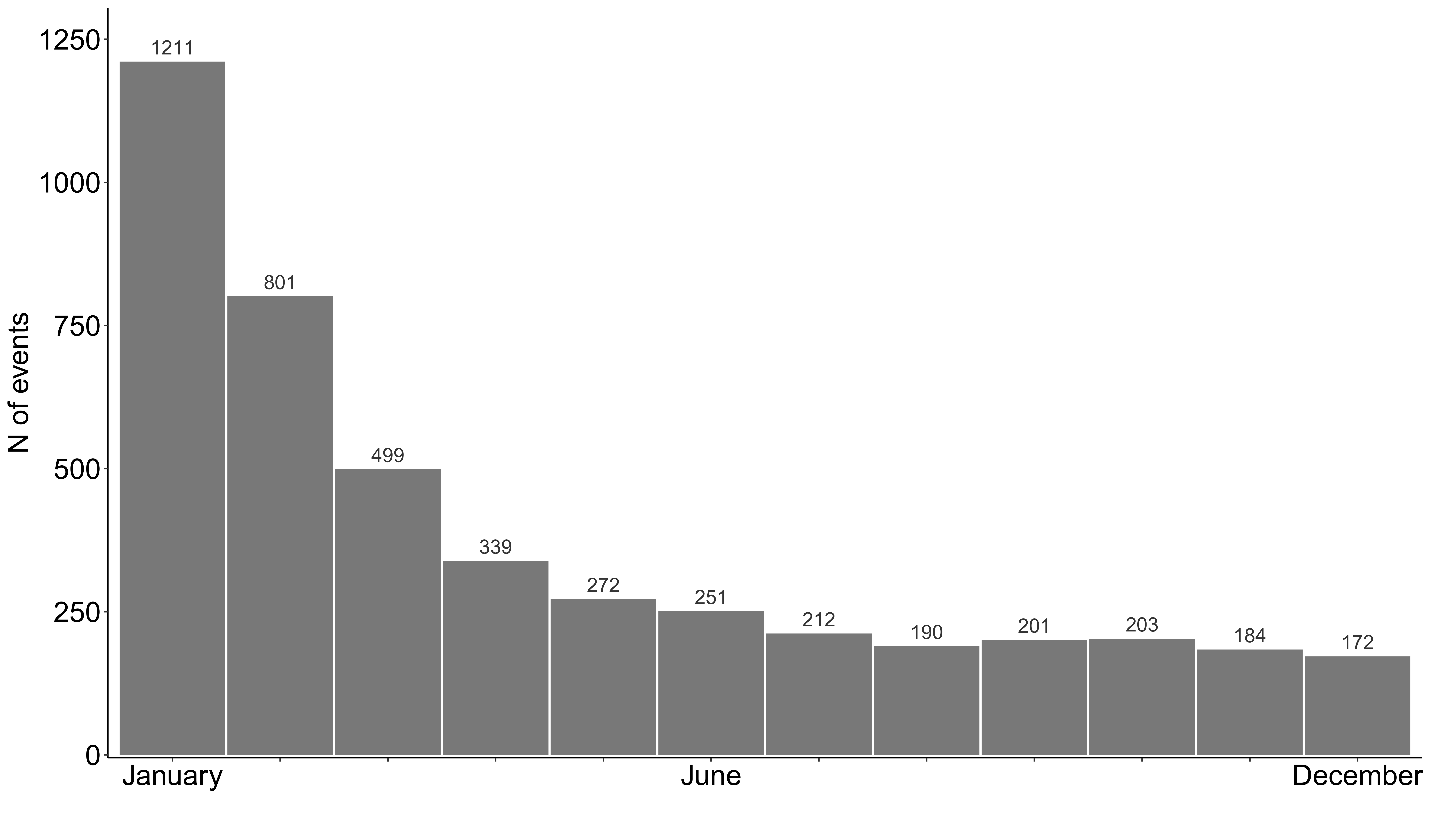


**Table S1.** Anatomic Therapeutic Chemical codes for Central nervous System-active medications.

| **Medication** | **ATC code** | **Medication** | **ATC code** |
| --- | --- | --- | --- |
| Antidepressants (class) | | Antipsychotics (class) | |
| Desvenlafaxine | N06AX23 | Chlorpromazine | N05AA01 |
| Mianserin | N06AX03 | Levomepromazine | N05AA02 |
| Trazodone | N06AX05 | Promazine | N05AA03 |
| Fluoxetine | N06AB03 | Triflupromazine | N05AA05 |
| Citalopram | N06AB04 | Fluphenazine | N05AB02 |
| Paroxetine | N06AB05 | Perphenazine | N05AB03 |
| Sertraline | N06AB06 | Trifluoperazine | N05AB06 |
| Fluvoxamine | N06AB08 | Periciazine | N05AC01 |
| Escitalopram | N06AB10 | Thioridazine | N05AC02 |
| Antiepileptics (class) | | Haloperidol | N05AD01 |
| Phenobarbital | N03AA02 | Bromperidol | N05AD06 |
| Carbamazepine | N03AF01 | Sertindole | N05AE03 |
| Valproic Acid | N03AG01 | Ziprasidone | N05AE04 |
| Phenytoin | N03AB02 | Zuclopenthixol | N05AF05 |
| Topiramate | N03AX11 | Lurasidone | N05AE05 |
| Levetiracetam | N03AX14 | Pimozide | N05AG02 |
| Oxcarbazepine | N03AF02 | Loxapine | N05AH01 |
| Zonisamide | N03AX15 | Clozapine | N05AH02 |
| Lamotrigine | N03AX09 | Olanzapine | N05AH03 |
| Lacosamide | N03AX18 | Quetiapine | N05AH04 |
| Gabapentin | N02BF01 | Asenapine | N05AH05 |
| Pregabalin | N02BF02 | Clotiapine | N05AH06 |
| Benzodiazepines (class) | | Quetiapine | N05AH04 |
| Clonazepam | N03AE01 | Risperidone | N05AX08 |
| Midazolam | N05CD08 | Aripiprazole | N05AX12 |
| Diazepam | N05BA01 | Paliperidone | N05AX13 |
| Oxazepam | N05BA04 | Iloperidone | N05AX14 |
| Clorazepate | N05BA05 | Brexpiprazole | N05AX16 |
| Lorazepam | N05BA06 | Antidepressants (class) | |
| Bromazepam | N05BA08 | Clomipramine | N06AA04 |
| Clobazam | N05BA09 | Amitriptyline | N06AA09 |
| Alprazolam | N05BA12 | Nortriptyline | N06AA10 |
| Opioids (class) | | Imipramine | N06AA02 |
| Tramadol | N02AX02 | Clomipramine | N06AA04 |
| Morphine | N02AA01 | Amitriptyline | N06AA09 |
| Oxycodone | N02AA05 | Nortriptyline | N06AA10 |
| Fentanyl | N02AB03 | Mianserin | N06AX03 |
| Buprenorphine | N02AE01 | Trazodone | N06AX05 |
| Nalbuphine | N02AF02 | Mirtazapine | N06AX11 |
| Codeine | N02AJ06 | Bupropion | N06AX12 |
| Z-drugs (class) | | Venlafaxine | N06AX16 |
| Zopiclone | N05CF01 | Duloxetine | N06AX21 |
| Zolpidem | N05CF02 | Agomelatine | N06AX22 |
| Eszopiclone | N05CF04 |  | |

**Table S2.** Positive predictive value of SNOMED CT terms based on auditing of random samples of 100 patients for each diagnosis.

| **Covariate** | **Positive predictive value^1^** |
| --- | --- |
| Alcohol abuse | 82.0 % |
| Alzheimer disease | 87.0 % |
| Anxiety | 88.0 % |
| Asthma | 36.0 % |
| Bipolar disease | 82.0 % |
| Cancer | 95.0 % |
| Cardiac failure | 76.0 % |
| Cirrhosis | 28.0 % |
| Chronic kidney disease | 87.0 % |
| Cognitive complaint | 94.0 % |
| Chronic obstructive pulmonary disease | 50.0 % |
| Coronary disease | 61.0 % |
| Dementia, frontotemporal | 85.3 % |
| Dementia, Lewy body | 79.0 % |
| Dementia, unspecified | 85.0 % |
| Dementia, vascular | 83.0 % |
| Depressive disorder | 80.0 % |
| Diabetes mellitus | 80.0 % |
| Epilepsy | 40.0 % |
| Hypertension | 97.0 % |
| Insomnia | 90.0 % |
| Parkinson’s disease | 80.0 % |
| Peripheral vascular disease | 78.0 % |
| Sleep disorder | 90.0 % |
| Schizophrenia | 86.0 % |
| Tobacco use, past or current | 76.0 % |

1. Defined as number of cases classified as true cases by medical doctors/cases captured by the SNOMED CT term.

**Table S3.** Characteristics of adults aged 60 and older, according to their Central Nervous System-active medication exposure on January 1^st^, 2021 (Hospital Italiano de Buenos Aires).

| **Characteristics** | **No CNS-active polypharmacy**  **(N= 62,646)** | | **CNS-active polypharmacy**  **(N=1,211)** | | **SMD^1^** |
| --- | --- | --- | --- | --- | --- |
|  | N | (%) | N | (%) |  |
| **Demographics** |  |  |  |  |  |
| Age group, in years |  |  |  |  |  |
| 60 to 69 | 18,048 | (29) | 235 | (19) | 0.22  0.16  0.34 |
| 70 to 79 | 22,423 | (36) | 344 | (28) |  |
| 80 or more | 22,175 | (35) | 632 | (52) |  |
| Female | 41,149 | (66) | 961 | (79) | 0.31 |
| **Mental Health diagnoses** |  |  |  |  |  |
| Anxiety disorder | 13,031 | (21) | 544 | (45) | 0.53 |
| Depressive disorder | 8,300 | (13) | 614 | (51) | 0.88 |
| Sleep disorder | 6,860 | (11) | 329 | (27) | 0.42 |
| Epilepsy | 770 | (1) | 59 | (5) | 0.21 |
| Bipolar disorder | 206 | (<1) | 69 | (6) | 0.32 |
| Schizophrenia | 61 | (<1) | <20 | (<1) | 0.13 |
| **Neurodegenerative diseases** |  |  |  |  |  |
| Cognitive complaint | 3,954 | (6) | 362 | (30) | 0.64 |
| Dementia, any type | 1,493 | (2) | 207 | (17) | 0.51 |
| Alzheimer’s disease | 308 | (1) | 43 | (4) | 0.22 |
| Vascular dementia | 94 | (<1) | <20 | (1) | 0.13 |
| Frontotemporal dementia | <20 | (<1) | <20 | (<1) | 0.10 |
| Lewy body dementia | 109 | (<1) | <20 | (1) | 0.15 |
| Parkinson’s disease | 463 | (1) | 29 | (2) | 0.13 |
| **Other comorbidities** |  |  |  |  |  |
| COPD | 2,909 | (5) | 121 | (10) | 0.21 |
| Asthma | 3,557 | (6) | 103 | (9) | 0.11 |
| Cancer | 7,606 | (12) | 196 | (16) | 0.12 |
| Hypertension | 40,303 | (64) | 842 | (70) | 0.11 |
| Cardiac failure | 2,694 | (4) | 116 | (10) | 0.21 |
| Coronary disease | 5,017 | (8) | 129 | (11) | 0.09 |
| Peripheral vascular disease | 1,386 | (2) | 41 | (3) | 0.07 |
| Diabetes mellitus | 7,513 | (12) | 172 | (14) | 0.07 |
| Chronic kidney disease | 2,996 | (5) | 79 | (7) | 0.08 |
| Cirrhosis | 686 | (1) | 26 | (2) | 0.08 |
| Alcohol use (past or current) | 787 | (1) | 32 | (3) | 0.10 |
| Tobacco use (past or current) | 13,135 | (21) | 373 | (31) | 0.23 |
| Hospital admissions in the previous year | |  |  |  |  |
| 1 admission | 4,551 | (7) | 232 | (19) | 0.17 |
| 2 admissions or more | 1,148 | (2) | 71 | (6) | 0.21 |

CNS: central nervous system; COPD: chronic obstructive pulmonary disease; SMD: standardized mean differences.

Frequencies lower than 20 are shown as inequalities to protect patient’s privacy.

1. Differences equal to or higher than 0.10 were considered significant.

**Table S4.** Individual drugs involved in 24,596 Central Nervous System-active polypharmacy events.

| **Medication** | **Frequency** | **(%)^1^** | **Medication** | **Frequency** | **(%)^1^** |
| --- | --- | --- | --- | --- | --- |
| 1. Clonazepam | 10,399 | (12.9) | 26. Bromazepam | 711 | (0.9) |
| 1. Quetiapine | 8,959 | (11.1) | 27. Levetiracetam | 679 | (0.8) |
| 1. Alprazolam | 5,232 | (6.5) | 28. Zopiclone | 535 | (0.7) |
| 1. Pregabalin | 5,073 | (6.3) | 29. Diazepam | 508 | (0.6) |
| 1. Escitalopram | 5,018 | (6.2) | 30. Codeine | 497 | (0.6) |
| 1. Zolpidem | 4,876 | (6.0) | 31. Topiramate | 460 | (0.6) |
| 1. Tramadol | 4,314 | (5.3) | 32. Carbamazepine | 445 | (0.6) |
| 1. Lorazepam | 3,504 | (4.3) | 33. Haloperidol | 394 | (0.5) |
| 1. Sertraline | 3,066 | (3.8) | 34. Aripiprazole | 355 | (0.4) |
| 1. Venlafaxine | 2,909 | (3.6) | 35. Morphine | 293 | (0.4) |
| 1. Risperidone | 2,697 | (3.3) | 36. Midazolam | 274 | (0.3) |
| 1. Mirtazapine | 2,370 | (2.9) | 37. Lacosamide | 120 | (0.1) |
| 1. Lamotrigine | 2,137 | (2.6) | 38. Phenobarbital | 91 | (0.1) |
| 1. Olanzapine | 1,691 | (2.1) | 39. Oxcarbazepine | 91 | (0.1) |
| 1. Trazodone | 1,454 | (1.8) | 40. Clotiapine | 86 | (0.1) |
| 1. Paroxetine | 1,431 | (1.8) | 41. Clozapine | 67 | (0.1) |
| 1. Citalopram | 1,372 | (1.7) | 42. Clomipramine | 53 | (0.1) |
| 1. Duloxetine | 1,248 | (1.5) | 43. Oxycodone | 33 | (<0.1) |
| 1. Amitriptyline | 1,238 | (1.5) | 44. Thioridazine | 28 | (<0.1) |
| 1. Eszopiclone | 1,211 | (1.5) | 45. Clorazepate | 27 | (<0.1) |
| 1. Levomepromazine | 1,093 | (1.4) | 46. Oxazepam | 25 | (<0.1) |
| 1. Valproate acid | 979 | (1.4) | 47. Zonisamide | 24 | (<0.1) |
| 1. Fluoxetine | 918 | (1.1) | 48. Sulpiride | 24 | (<0.1) |
| 1. Gabapentin | 903 | (1.1) | 49. Clobazam | 21 | (<0.1) |
| 1. Desvenlafaxine | 834 | (1.0) | 50. Others | 6 | (<0.1) |

1. Percent calculated over a total of 80,775 central nervous system-active medications prescriptions involved in 24,596 central nervous system-active polypharmacy events among 4,535 patients. Multiple Central Nervous System-active polypharmacy episodes were allowed per patient. Percents may not add exactly 100% due to rounding.
